# Optimizing imaging in orbital vascular anomalies: a review on matching modality to pathology for effective diagnosis and treatment planning

**DOI:** 10.3389/fopht.2026.1730495

**Published:** 2026-04-13

**Authors:** Arshia Arjomandi, Katherine M. Lucarelli, Robert A. Goldberg, Daniel B. Rootman

**Affiliations:** Department of Orbital and Ophthalmic Plastic Surgery, Jules Stein Eye Institute, University of California, Los Angeles, CA, United States

**Keywords:** arteriovenous malformation, imaging modalities, lymphatic-venous malformation, orbital vascular anomalies, spatial resolution, temporal resolution, treatment planning, vascular malformations

## Abstract

**Introduction:**

Orbital vascular anomalies (OVAs) encompass a heterogeneous group of lesions requiring precise imaging to guide diagnosis and treatment. Multiple imaging modalities offer distinct strengths and limitations, with the choice guided by the balance between spatial and temporal resolution. This review aligns imaging modalities with specific OVM subtypes to optimize diagnostic accuracy and procedural planning, while highlighting advanced and evolving imaging techniques that may further enhance clinical decision-making.

**Methods:**

A narrative review was conducted on studies describing imaging characteristics, diagnostic performance, and clinical utility in OVAs, supplemented with imaging from patients presenting with a wide range of lesions. All patient data were collected and reviewed in compliance with HIPAA regulations and international ethical standards.

**Results:**

Although ultrasound provides dynamic assessment of vascular flow and superficial morphology, its role is limited in modern practice due to poor spatial resolution and depth penetration. CT offers superior spatial resolution for osseous and calcified lesions, while MRI provides excellent soft tissue characterization and evaluation of complex low-flow malformations. MR and CT angiography deliver detailed vascular mapping critical for pre-embolization planning, yet their static nature limits evaluation of dynamic changes. Dynamic techniques, such as Time-Resolved Imaging of Contrast KineticS (TRICKS) MRI angiography and dynamic CT angiography, enable real-time assessment of flow and venous distensibility, improving procedural planning. Conventional digital subtraction angiography remains the standard for complex lesions in critical locations, combining high-temporal-resolution diagnosis with therapeutic intervention. Given the heterogeneity of OVAs, a multimodal approach is often necessary to address diagnostic, planning, and treatment needs comprehensively.

**Conclusion:**

Optimal imaging of OVAs requires tailoring modality selection to lesion type and clinical context. Incorporating advanced and emerging imaging approaches into clinical practice may further improve diagnostic precision, procedural planning, and patient outcomes.

## Introduction

Imaging is fundamental in the management of vascular anomalies. Using a wide array of tools, imaging plays an important role in diagnosis, treatment planning and in some cases, direct management. When selecting an imaging modality for evaluating vascular anomalies, optimizing spatial and temporal resolution is a key consideration. Spatial resolution refers to ability to distinguish fine structural detail, which is important in defining lesion boundaries and relationships to local structures (1). In contrast, temporal resolution describes capacity to capture rapid changes over time, which is useful in understanding the vascular dynamics of inflow and outflow. Utilizing modalities with high temporal resolution is particularly important in real-time procedural settings, such as during embolization or sclerotherapy, where intra-procedural vascular changes directly influence outcomes (2).

In this paper, we outline key classification systems for vascular anomalies and examine the major imaging modalities used in their evaluation, including ultrasound, computed tomography (CT)/computed tomography angiogram (CTA), magnetic resonance Imaging (MRI)/magnetic resonance angiogram (MRA), dynamic dual phase techniques, and conventional angiography. We describe these modalities in terms of trade offs between spatial and temporal resolution. Based on these features, we expand on the diagnostic utility and role in treatment planning for each. Understanding how each imaging technique aligns with specific clinical goals is essential for selecting the most appropriate modality and delivering effective, individualized care.

## Classification of orbital vascular anomalies

Orbital vascular anomalies (OVAs) represent a diverse spectrum of lesions within the orbit, with several classification systems in use. The International Society for the Study of Vascular Anomalies (ISSVA) classification, developed by Mulliken and Glowacki in the 1980s, divides vascular anomalies based on endothelial proliferative activity into tumor and malformation groups (3, 4). Vascular tumors are distinguished by their nature as cellular proliferations and are subdivided into a range of benign, locally aggressive and malignant lesions. Malformations, in contrast, represent congenital structural anomalies and, as emphasized in the 2025 ISSVA update, are vascular anomalies largely driven by somatic genetic mutations that disrupt vascular development rather than endothelial proliferation. They may be simple, involving a single vascular component (arterial, venous, lymphatic), or complex, comprising multiple constituents such as venolymphatic or arteriovenous elements (4, 5). In 2014, Rootman adapted this classification to orbital vascular anomalies, where the flow physiology and lymphatic morphology are perhaps more critical for defining intervention (6). This classification maintains the division of vascular malformations into high-flow and low-flow categories, further subcharacterizing venous lesions as distensible or nondistensible based on their expansion with Valsalva maneuver, and additionally classifying lymphatic malformations as microcystic or macrocystic (3).

The following sections examine the key imaging modalities used in vascular evaluation. Each modality is discussed based on its spatial and temporal resolution, diagnostic utility, and role in treatment planning across different vascular lesions.

## Ultrasound

Ultrasound imaging is distinguished by its excellent temporal resolution and specialized Doppler capabilities. High temporal resolution allows dynamic, real-time analysis of vascular spaces. Doppler ultrasound can provide information about blood flow velocity and direction which is particularly useful for identifying feeder vessels and arteriovenous shunting in high-flow lesions like AVMs (7, 8). The modality is non-invasive and does not subject patients to ionizing radiation. It additionally requires less specialized equipment and can be performed dynamically in a clinical setting. The favorable safety profile, lack of radiation exposure, cost-effectiveness, and ease of use make it a useful diagnostic tool, particularly in pediatric patients requiring multiple evaluations (9, 10).

**Figure 1:**
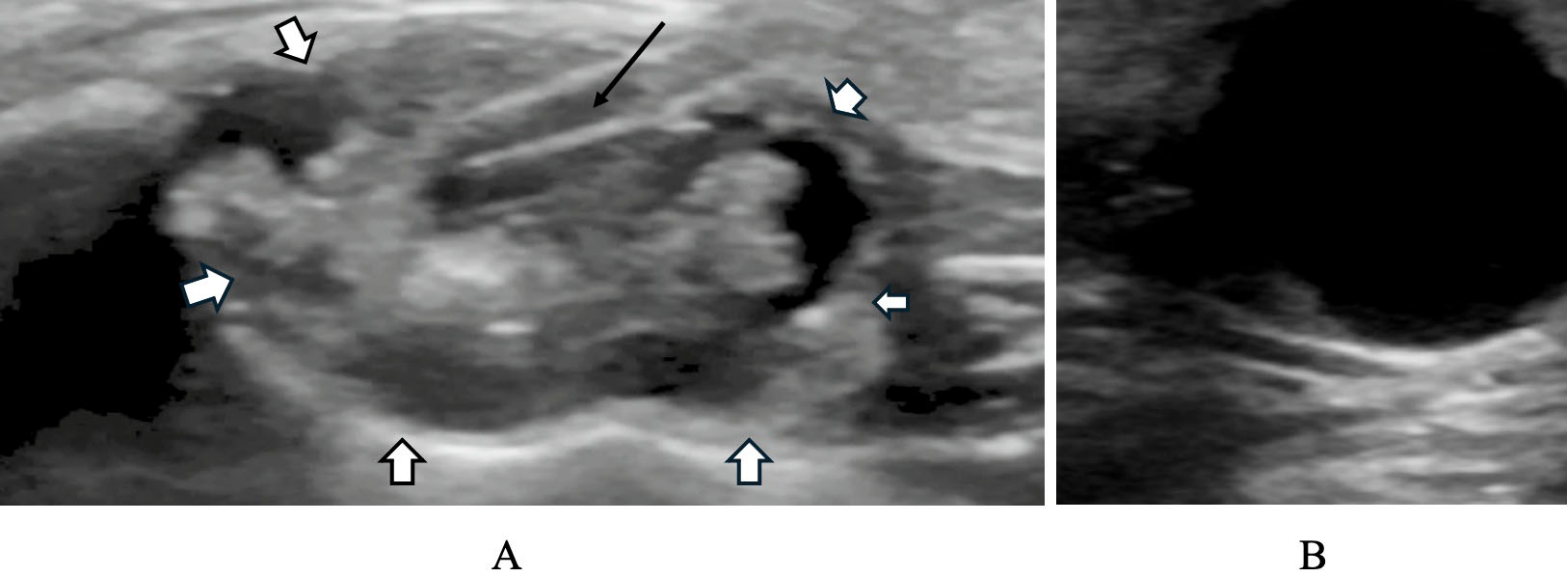
Ultrasound imaging of cystic orbital lesions. **(A)** Ultrasound-guided needle aspiration of a macrocystic lesion. The needle (arrow) is visualized as an echogenic linear structure entering the cyst cavity, with the surrounding cyst wall visible as a thin hyperechoic margin (arrow heads). **(B)** Ultrasound showing a well−defined, anechoic cystic space within the orbit with smooth internal borders.

Ultrasound may be applied in the evaluation and monitoring of periorbital and orbital infantile hemangiomas, offering a radiation and sedation free option for repeat assessments (9, 10). It also plays a role in both diagnosis and management of macrocystic lymphatic malformations (LMs). These can be visualized as hyperechoic walled, anechoic cysts (though they can have variably echoic contents). In the dynamic setting ultrasound scan can be used to localized treatment placement in aspiration and sclerotherapy (**Figure 1**) (10). Doppler ultrasound is useful in the evaluation of high-flow AVMs and differentiating them from venous and lymphatic malformations (10).

Ultimately, ultrasound is limited by relatively low spatial resolution and limited tissue penetrance, restricting its utility primarily to superficial or anterior lesion. Its reduced ability to resolve deeper anatomical structures or complex soft tissue relationships makes it less suitable for evaluating deeper or complex orbital anomalies (11). Ultrasound imaging can also be affected by motion artifacts and operator dependence, which may complicate evaluation in uncooperative patients.

## Conventional computed tomography

**Figure 2:**
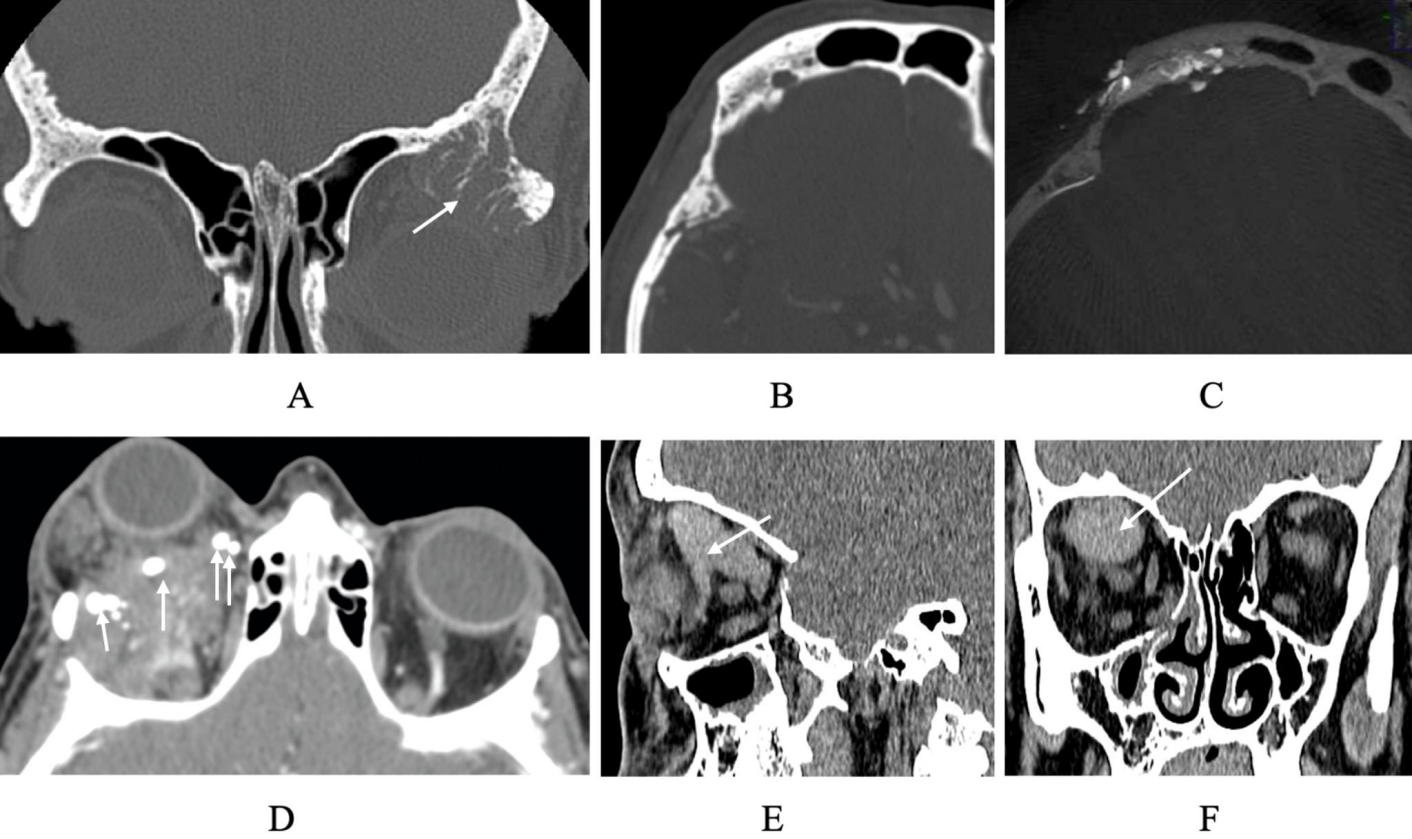
CT imaging of orbital vascular anomalies. **(A)** Coronal CT showing a left superolateral orbital rim intraosseous hemangioma (arrow), characterized by coarse trabeculations and expansion of the involved bone. **(B)** Axial CT demonstrating temporalis fossa remodeling and flow voids within the frontal bone due to involvement with VM **(C)** same frontal bone intraosseous VM with embolic material in vascular channels. **(D)** Axial CT demonstrating a right orbital complex venous–lymphatic malformation containing multiple phleboliths, seen as round hyperdense calcifications within the lesion (arrows) **(E)** Sagittal and coronal **(F)** views of large SOV thrombosis (F arrow) with involvement of lateral collateral vein (E arrow).

Conventional CT offers excellent spatial resolution, making it useful for evaluating detailed orbital anatomy. It is useful for elucidating highly dense structures such as bone and other calcium depositions, including phlebolith (12, 13). In cases involving previous surgery, CT is also valuable for localizing metallic implants or clips. Additionally, it is considered the preferred modality for assessment of hemangioma of bone, which demonstrate a distinctive, pathognomonic “corduroy” appearance characterized by internal trabeculations with a sunburst appearance (**Figures 2A–C**) (14, 15).

CT is useful in the assessment of several orbital vascular anomalies (16, 17). Lymphatic malformations often appear as grape-like, non-enhancing cystic clusters with internal septations, while LVMs present as poorly defined, mildly enhancing lesions that often cross anatomical boundaries (**Figures 2D–F**) (16, 17). CT is also used to evaluate cavernous venous malformations, which appear as well-circumscribed, ovoid hyperdense with smooth borders and have variable internal consistency (18). Hemangioma of bone, as noted, are more clearly visualized on CT due to their density and osseous involvement (**Figure 2A**) (5, 18, 19).

The primary limitations of conventional CT are poor temporal resolution and limited soft tissue differentiation. Although CT can indicate blood flow within lesions, particularly capillary patterns, it produces static images and cannot capture real-time physiological changes, making it unsuitable for dynamic assessments. Additionally, CT offers limited soft tissue contrast, reducing its accuracy in evaluating complex soft tissue lesions or thrombosis. Radiation exposure also makes it a less ideal choice for pediatric patients or repeated follow-up imaging. Furthermore, overlapping imaging characteristics of low-flow lesions on CT can lead to diagnostic ambiguity, often necessitating correlation with other modalities for definitive characterization (17).

## Conventional magnetic resonance imaging

**Figure 3:**
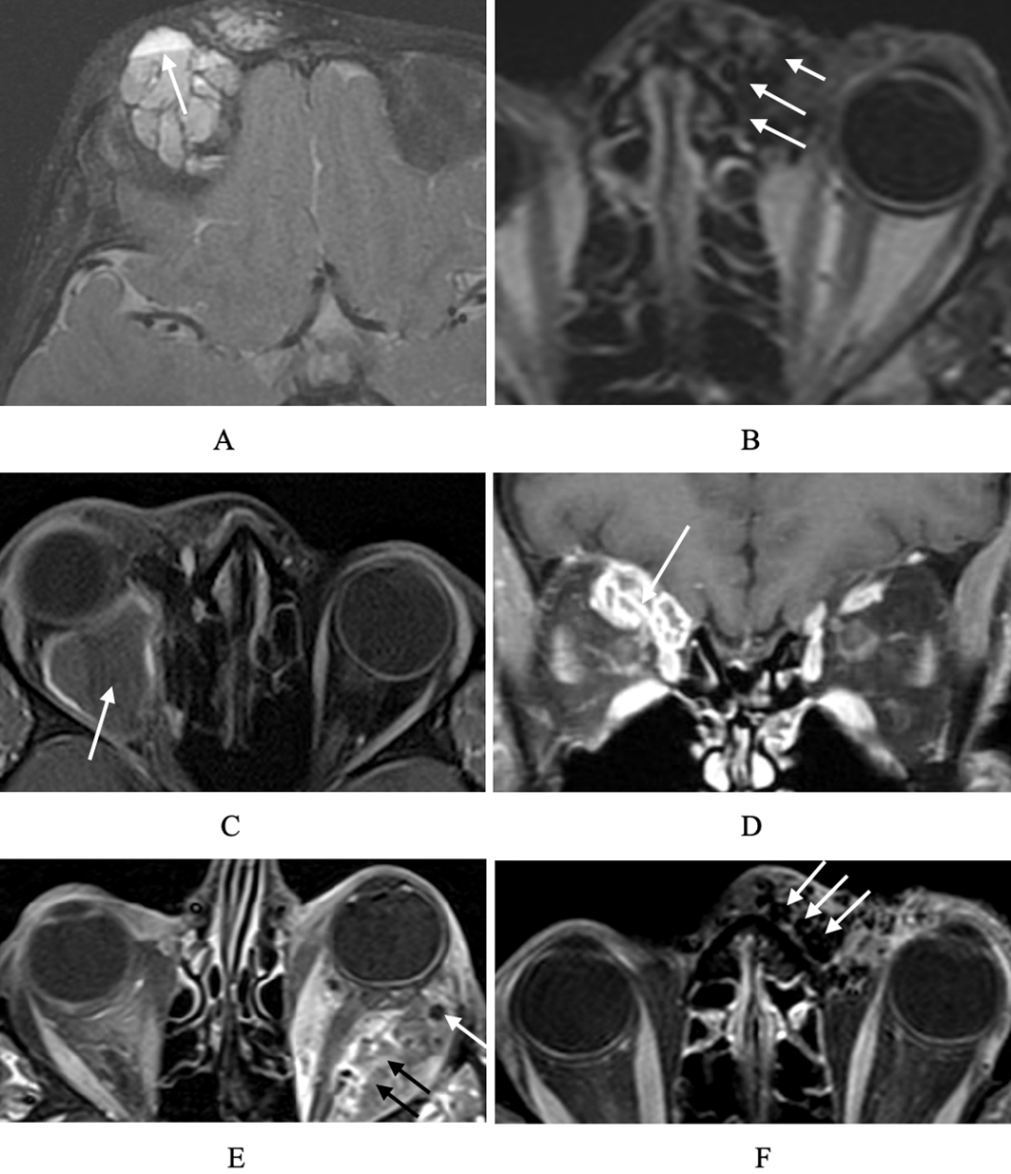
MRI imaging of orbital vasculature. **(A)** Axial MRI demonstrating fluid–fluid levels (arrow), visible as horizontal interfaces between hyperintense and hypointense components within a multiloculated cystic space. **(B)** Axial MRI showing flow voids (arrows), identified as round or tubular areas of signal absence consistent with rapidly flowing blood. **(C)** T1 contrast enhanced sequence of large intraconal VLM with thrombosis demonstrating peripheral enhancement and biphasic internal consistency characteristic of degrading blood products and **(D)** coronal T1 fat saturated MRI with contrast demonstrating extraconal multiseptated VLM with thrombosis, which appears as pockets of hypointense non-enhancing lumen with enhancement of the vessel wall septations (arrow). **(E)** MRI of a lymphatic–venous malformation corresponding to **Figure 2D**, with dark spaces representing phlebolith (white arrow) and multiloculated microcystic spaces (black arrows). **(F)** MRI demonstrating an arteriovenous malformation, distinguished by serpiginous vascular channels with prominent flow voids reflecting high-flow arterialized blood (arrows).

MRI provides excellent spatial resolution and soft tissue contrast, making it the preferred modality for evaluating a wide array of orbital pathologies, including OVA (**Figure 3**). A range of sequences can be utilized and specific information gleaned from each. Flow voids and phlebolith can be visualized in negative space as dark structures on both T1 and T2 (**Figure 3B**). Cysts present as round or oval structures with enhancing peripheral walls and central regions of varying T2 intensity depending on the water and protein content contained within. Thrombosis can be visualized, and characterized in terms of age based on the relative intensity on T1 and T2 imaging (**Figures 3C, D**).

High spatial resolution allows for identification of soft tissue elements and relationships to local structures including the extraocular muscles and the optic nerve. Communication between the orbit and regional spaces including the intracranial and sinus cavities can be noted. Although primarily a diagnostic tool, MRI also contributes to treatment planning by delineating lesion extent, guiding surgical or interventional approaches, and identifying lymphatic components relevant for therapy, including sclerotherapy or biologics. High-resolution soft tissue detail also supports accurate postoperative follow-up.

MRI is optimal for evaluation of complex low-flow anomalies such as lymphatic malformations (LMs) and lymphatic-venous malformations (LVMs), where it excels in delineating cystic architecture and vascular components (**Figures 3E, F**) (16, 20). It can also be utilized in conjunction with MR angiography in the assessment of arteriovenous malformations (AVMs) to evaluate soft tissue involvement and to identify enlarged vascular elements that may serve as vessel conduits for intravascular therapy or targets for transcutaneous embolization (21).

Despite its strengths, MRI is less effective for evaluating bone and calcified lesions. Metallic implants can cause significant susceptibility artifacts, compromising image quality in postoperative patients. Furthermore, MRI’s temporal resolution is very low as each sequence usually requires more than 5 minutes to acquire, limiting the use of conventional MRI in dynamic vascular evaluation.

## MR or CT angiogram

**Figure 4:**
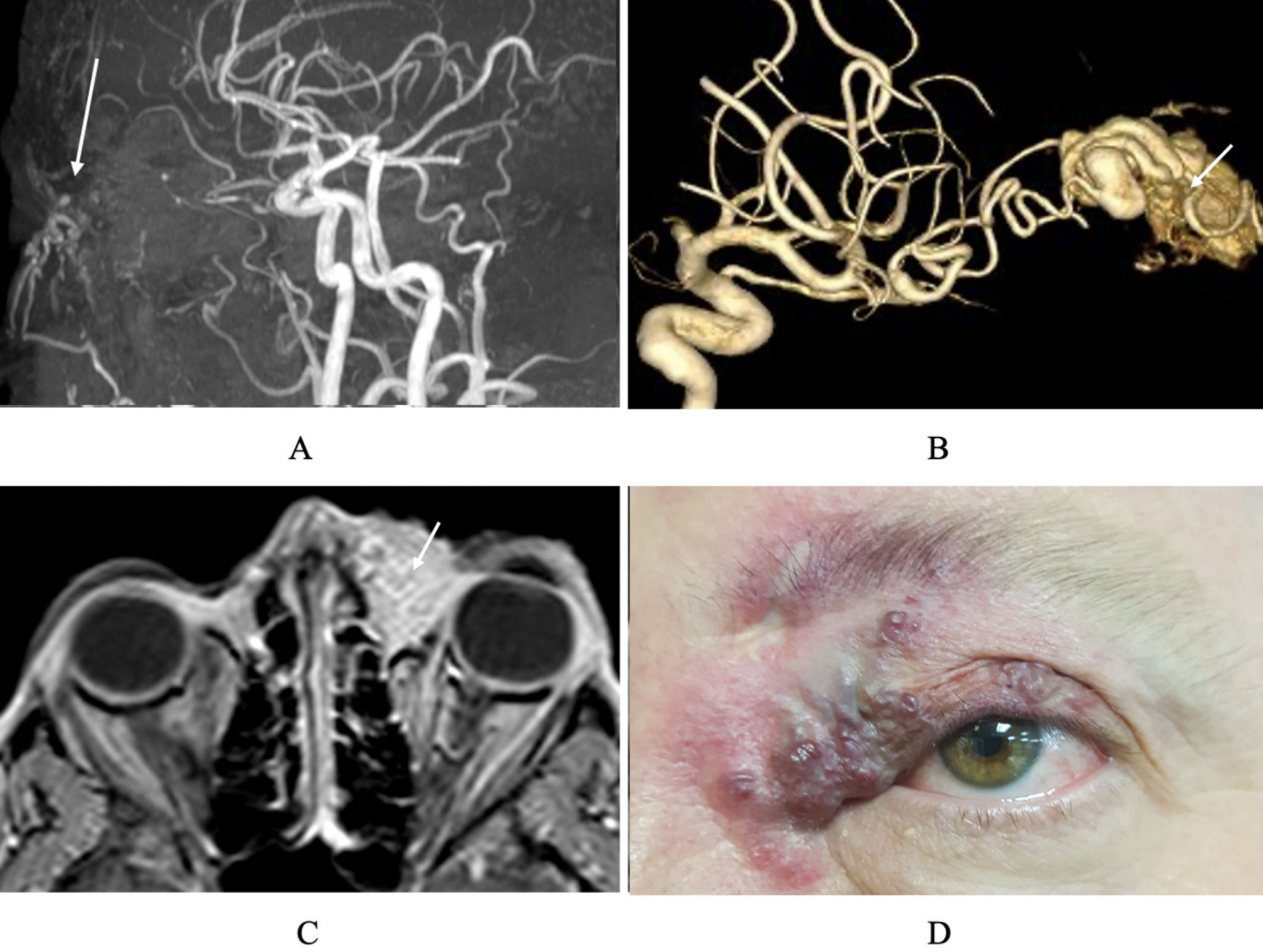
MR and CT angiography of orbital and cerebral vasculature. **(A)** MR angiogram sagittal view demonstrating medial canthal AVM. **(B)** CT angiogram with 3D reconstruction showing tortuous arterial feeder vessels and a nidus (arrow), consistent with AVM. **(C)** T1 MRI with contrast demonstrating AVM located in the anteromedial left orbit, characterized by diffuse contrast enhancement **(D)** External photograph demonstrating anteromedial orbital AVM.

MR angiography provides excellent spatial resolution, making it well-suited for detailed visualization of vascular anatomy (**Figures 4A–C**). These can be helpful in defining the morphology of arterial lesions such as arteriovenous malformations (AVMs) and fistulas (22). CT angiography (CTA) is similar in utility though more rapid in acquisition. These techniques allow for identification of vascular architecture and are utilized in treatment planning (**Figure 4D**). MRA may be advantageous for patients requiring radiation-free assessment (e.g., pediatric or serial evaluations) (22, 23).

Despite strengths in spatial detail, both CT and MR angiography demonstrate poor temporal resolution. They offer static snapshots of the vascular anatomy and fail to capture information regarding flow dynamics. As a result, they are limited in assessing physiologic flow patterns, venous distensibility, or dynamic vascular changes (e.g., with Valsalva). These constraints make them suboptimal for evaluating venous malformations that benefit from phase-based imaging. In addition, contrast safety must be considered, as iodinated agents used for CT angiography can affect renal function and may trigger allergic reactions, whereas gadolinium-based agents for MR angiography, though generally well tolerated, have been linked to rare cases of nephrogenic systemic fibrosis in patients with significant kidney impairment (23).

## Dynamic MRI angiogram

**Figure 5:**
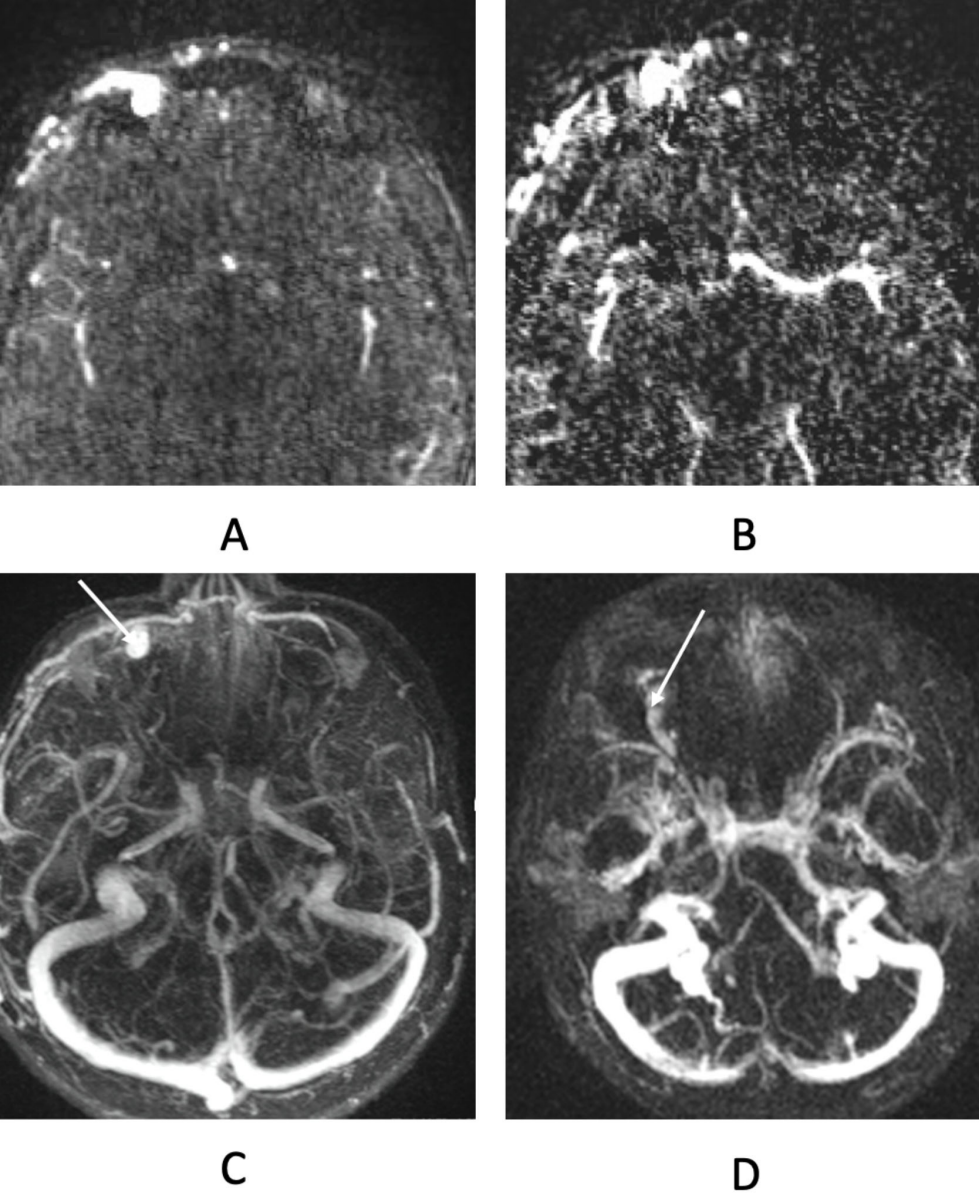
Dynamic MR angiography (TRICKS-MRA) showing sequential contrast enhancement and vessel subtraction. **(A)** Individual slice of full head MRI taken at 39 seconds demonstrating contrast in vessels. **(B)** Same slice of MRI viewed with tissue subtracted and contrast in vessels visualized. **(C)** Composite of all subtracted slices in MRI taken at 39 seconds demonstrating left eyelid arteriovenous malformation (AVM) on early-phase filling with dilated venous outflow (arrow). **(D)** late phase filling of VLM (arrow) in superolateral orbit.

Dynamic MRI angiography, for instance Time-Resolved Imaging of Contrast KineticS (TRICKS), is a noninvasive technique that provides a high temporal resolution MRI alternative. These sequences acquire a full head scan every 1.5–2 seconds and by digital subtraction, the vascular elements are highlighted. The scans are then overlayed in temporal sequence allowing for an assessment of the vasculature over time as contrast is injected (**Figures 5A–D**) (23).

This modality is valuable for evaluating arteriovenous malformations (AVMs) and other high flow malformations, as it provides both vascular anatomic detail (e.g. vessel density, lesion structure, and identification of feeding arteries, nidus architecture, and draining veins), and physiologic insight (e.g. timing and sequence of arterial inflow or venous filling) (**Figures 5C, D**) (23, 24). These sequences can be useful in planning optimal access routes in advance of embolization and/or sclerotherapy procedures (24).

The major limitation of dynamic MRI angiography is poor spatial resolution compared to conventional MRI or CT. Additionally, it is less effective in assessing venous outflow, as gadolinium contrast tends to linger within venous structures without clear washout, making drainage pathways harder to delineate (25). Consequently, this modality is not ideal for evaluating lesions with predominantly venous or lymphatic drainage.

## Dual phase CT angiogram

**Figure 6:**
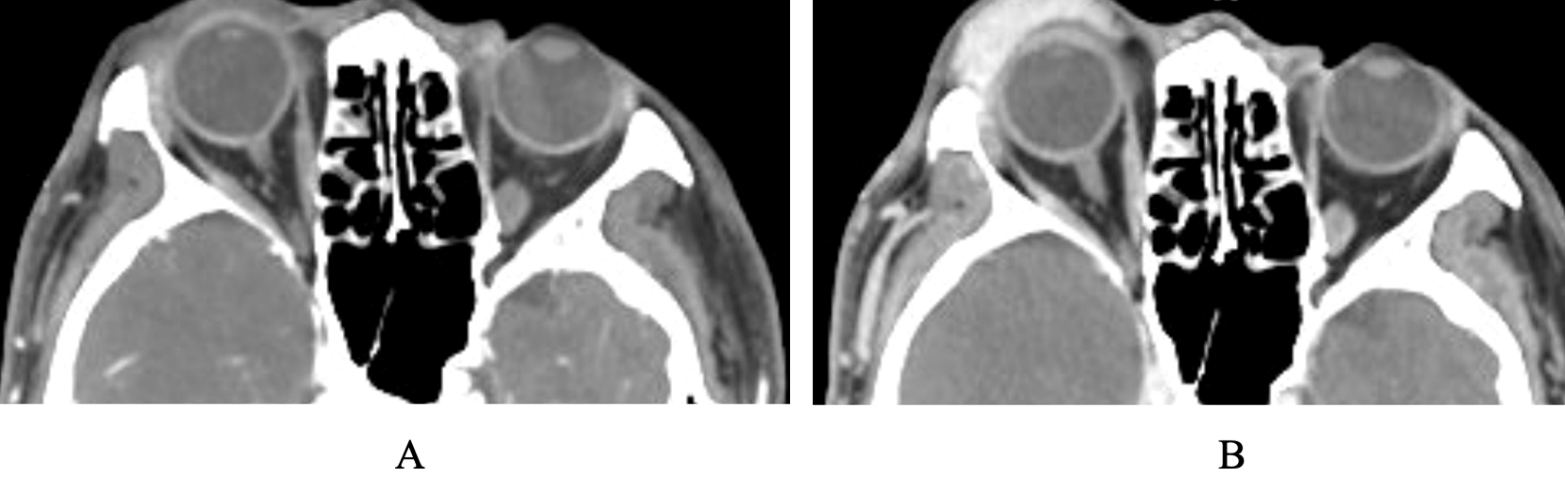
Dynamic CT angiography with Valsalva demonstrating a distensible venous malformation (VM) in the anterior right orbit. **(A)** Pre-Valsalva shows baseline size, almost imperceptible. **(B)** Post-Valsalva demonstrates lesion enlargement, consistent with venous distensibility.

Dynamic CT angiography, performed in this context as a dual-phase protocol, offers a mix of acceptable spatial and temporal resolution. The standard protocol involves an initial contrast-enhanced scan, followed by a second acquisition at approximately 70 seconds, often performed during a Valsalva maneuver, to assess changes in venous distension and tissue displacement (**Figures 6A, B**) (25). This dual-phase approach captures the mid-to-late venous phase, allowing characterization of lesion distensibility, venous outflow, and anatomic relationships within the orbit.

Dynamic CT angiography is useful for evaluating distensible venous malformations and slow-flow, non-distensible venous malformations as it provides valuable information regarding physiologic features, such as expansion with Valsalva and outflow behavior (25, 26). Slow-flow, non-distensible lesions (cavernous venous malformations) demonstrate progressive contrast enhancement, characterized by focal high-intensity early filling followed by diffuse, moderate-intensity late filling, without expansion during the Valsalva maneuver (27, 28). These features differentiate them from neoplasms like schwannoma, which present as well-circumscribed soft-tissue masses with contrast enhancement that does not change over time (28). This modality also supports diagnostic assessment by distinguishing between arterial and venous phases and may inform whether transvenous, transarterial or direct transcutaneous access is most appropriate for intervention (29).

The technique is limited by its temporal resolution, as it captures only two time points, unlike real-time flow visualization offered by MR or catheter angiography. Additionally, it involves ionizing radiation and iodinated contrast, making it less suitable for repeated imaging, individuals with kidney dysfunction and those allergic to contrast medium.

## Conventional endovascular angiography

**Figure 7:**
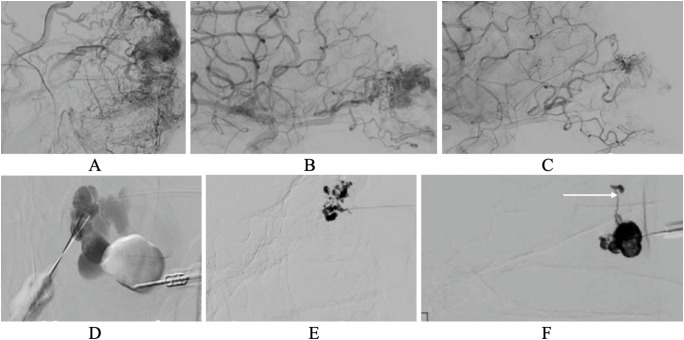
Pre- and post-embolization digital subtraction angiography (DSA) of an arteriovenous malformation (AVM) and examples of venous–lymphatic malformation (VLM) subtypes. **(A)** AVM showing a dense vascular nidus with early arteriovenous shunting. **(B)** Pre-embolization DSA demonstrating the nidus before intervention. **(C)** Post-embolization DSA demonstrating reduced nidus opacification following transarterial embolization. **(D)** Macrocystic VLM with multiple large contrast-filled cystic spaces, two percutaneous cannulae visible entering the lesion in different cysts. **(E)** Microcystic VLM filling with embolic material. **(F)** Distensible VLM showing expansion during contrast injection, with a needle/cannula positioned for percutaneous treatment. Feeder vessel retrograde filled (arrow).

Conventional angiography or digital subtraction angiography (DSA) is the gold standard for real-time vascular imaging due to its unmatched temporal resolution despite by definition offering only limited soft tissue spatial resolution. DSA acquires continuous X-ray images at 2–3 frames per second, enabling dynamic evaluation of intravascular anatomy, blood flow patterns, venous drainage characteristics, and physiologic changes during Valsalva maneuver (5, 30). Digital subtraction techniques enhance vascular contrast by removing background structures such as bone and soft tissue, providing clearer visualization of vascular architecture. DSA is the preferred modality for evaluating arteriovenous malformations (AVMs), including identification of the nidus and mapping of feeder and draining vessels, as well as a valuable tool diagnosis of arterovenous fistulae (**Figure 7**) (5, 21, 31).

In addition to diagnosis, conventional angiography plays a central role in treatment. It enables direct vascular access for embolization, sclerotherapy, and retrograde catheterization during the same session as diagnostic imaging (**Figures 7A–C**) (32–34). DSA is performed in real time, providing immediate feedback on vessel response and treatment efficacy.

While temporal resolution is excellent, the spatial resolution of conventional angiography is poor. It is limited in assessing extravascular anatomy, including adjacent soft tissues and bony structures, and may not fully capture the extent of complex lesions beyond the vascular lumen. Additionally, this technique is invasive, requiring arterial or venous access, and carries risk of complications such as cerebral infarction and ophthalmic artery thrombosis (23). It also involves radiation exposure, making it less favorable than noninvasive imaging options in certain clinical scenarios.

## Discussion

In the evaluation and management of orbital vascular anomalies, the appropriate imaging modality optimizes a balance of spatial and temporal resolution according to clinical needs. Spatial resolution emphasizes structural detail, whereas temporal resolution captures dynamic, real-time vascular changes essential for functional assessment and guidance in selecting appropriate intervention. Based on these considerations, each imaging technique demonstrates unique strengths and limitations, making their selection context dependent. See imaging modalities for orbital vascular anomalies summarized below in Table 1.

**Table 1:** Summary of imaging modalities for orbital vascular anomalies.

Modality	Spatial resolution	Temporal resolution	Course of treatment	Lesion types
Ultrasound	Poor	Excellent	Diagnosis	- Anterior cystic lesions - Macrocystic LM - Infantile Hemangioma - High flow AVM
Conventional CT	Good	Poor	Diagnosis	- LM and LVMs (e.g. with phleboliths) - Cavernous and Intraosseous Hemangiomas - Orbital fractures and Bone remodeling
Conventional MRI	Excellent	Poor	Diagnosis	- Low flow LM and LVM - Thrombosis (SOVT, varices) - Hemorrhage and fluid-fluid levels - Orbital tumors and complex soft tissue lesions
MR/CT Angiogram	Good	Poor	Planning	- Arterial pathologies: AVM and Fistula
Dynamic MRI Angiogram	Poor	Good	Planning	- AVM - LVM (arterial phase)
Dynamic CT Angiogram	Good	Good	Planning	- Distensible VM (Valsalva)
Conventional Angiography	Poor	Excellent	Assessment and Management	- AVM (nidus, feeder/drainer mapping) - LVM (especially distensible) - AV Fistula - Real-time embolization & sclerotherapy guidance

LM, lymphatic malformation; LVM, lymphatic-venous malformation; SOVT, superior ophthalmic vein thrombosis; AVM, arteriovenous malformation; VM, venous malformation; AV fistula, arteriovenous fistula.

Ultrasound maintains high temporal resolution and allows for Doppler assessment of flow but has low spatial resolution and limited penetration. It can be useful thus in superficial anterior lesions, particularly those demonstrating significant echoic borders (cystic and vascular structures). Although ultrasound may not be the primary modality for most orbital vascular anomalies, it is useful for initial imaging in pediatric patients, given its safety profile, lack of radiation, and convenience for repeat imaging.

Noninvasive imaging, including static or dynamic CT or MRI, is more commonly utilized for the initial evaluation of lesions (3). Conventional CT provides good spatial resolution and enhances visualization of bony anatomy and calcified structures. MRI is similar, though maintains superior soft tissue resolution. Various MRI sequences can provide additional information regarding the cellular and acellular components of vascular lesions, including layering in macrocysts and temporal changes after hemorrhage. Both modalities are primarily limited by poor temporal resolution restricting utility in dynamic lesion assessment.

MR and CT angiography both provide detailed vascular maps valuable in pre-embolization planning. Despite high spatial resolution, these techniques represent a single snapshot in time and offer poor temporal resolution, limiting their effectiveness in evaluating dynamic vascular changes.

Dynamic non-invasive imaging modalities, such as Time-Resolved Imaging of Contrast KineticS (TRICKS) MRI angiography and dynamic CT angiography, offer a different balance of temporal and spatial resolution providing good temporal at the expense of spatial resolution (3). Time resolved MRI sequences allow for dynamic assessment of arterial flow, though due to staining of the endothelium limited information can be gathered regarding venous outflow. Dual phase CT angiography provides a two-phase picture of vascular flow, and in combination with Valsalva, and is useful in assessing venous components and their distensibility.

Finally, conventional angiography (DSA), remains the gold standard for complex lesions in critical locations. It combines diagnostic utility, real-time procedural imaging, and therapeutic intervention within a single modality, enabling precise monitoring during embolization and/or sclerotherapy.

In cases of complex combined vascular malformations, it may be necessary to utilize multiple imaging modalities. Each technique can provide specific and complementary information useful in understanding different aspects of the lesion in terms of morphology and physiology (3, 35). Due to the typically heterogeneous nature of OVAs, often no single modality is sufficient for comprehensive evaluation. This reinforces the importance of a tailored, multimodal imaging approach to optimize both diagnosis and treatment planning (35). Understanding both the nature of the disease and the qualities of the imaging modalities available allow for accurate matching of modality to pathology and further selection of appropriate treatment strategies.
